# Different goals, common ground: A global perspective on dairy breeding objectives

**DOI:** 10.3168/jdsc.2026-1009

**Published:** 2026-03-27

**Authors:** D.P. Berry, F. Miglior, C. Schmidtmann, K. Byskov, H. Splittorff, M. Rocland, R. Finocchiaro, G. De Jong, J. Cole, J. Graham, A. Barenco, T.F.O. Neuenschwander, M. Winters, P. Amer, M. Axford, K. Wijnrocx, N. Gengler, B.E. Mostert, M. Kelleher, J.A. Jiménez-Montero, O. Gonzalez-Recio, K. Potočnik, E. Ezra, J. Weller, L. Bognár, K. Rzewuska, M. Pszczola, T. Osawa, I. Aguilar, R.D. López-Correa

**Affiliations:** 1 Teagasc, Animal & Grassland Research and Innovation Centre, Moorepark, Fermoy, Co. Cork, P61 P302, Ireland; 2 Centre for Genetic Improvement of Livestock, Department of Animal Biosciences, University of Guelph, Guelph, ON, N1G 2W1, Canada; 3 Vereinigte Informationssysteme Tierhaltung w.V. (vit), 27283 Verden, Germany; 4 The Nordic Cattle Genetic Evaluation, DK-8200 Århus N, Denmark; 5 Idele, Institut de l'Élevage, 75012 Paris, France; 6 Associazione Nazionale Allevatori della Razza Frisona, Bruna e Jersey Italiana, Via Bergamo, 292 26100 Cremona, Italy; 7 Cooperative CRV u.a., 6842 BD Arnhem, the Netherlands; 8 Council on Dairy Cattle Breeding, Bowie, MD 20716; 9 Holstein Association USA, Brattleboro, VT 05302-0808; 10 Swissherdbook, CH-3052 Zollikofen, Switzerland; 11 Holstein Switzerland, 1730 Posieux, Switzerland; 12 Agriculture and Horticulture Development Board, Coventry, CV3 4PE, United Kingdom; 13 AbacusBio Ltd., Cargill House, Dunedin 9016, New Zealand; 14 DataGene, Bundoora, VIC 3083, Australia; 15 TERRA Teaching and Research Center, University of Liège, Gembloux Agro-Bio Tech (ULiège-GxABT), 5030 Gembloux, Belgium; 16 SA Stud Book, Bloemfontein, 9300, South Africa; 17 Irish Cattle Breeding Federation, Ballincollig, County Cork, P31 D452, Ireland; 18 Conafe, 28320 Pinto, Madrid, Spain; 19 The Roslin Institute and Royal (Dick) School of Veterinary Studies, Easter Bush Campus, Midlothian EH25 9RG, United Kingdom; 20 Department of Animal Science, University of Ljubljana, 1000 Ljubljana, Slovenia; 21 Israel Cattle Breeders' Association, Caesarea Industrial Park, 3079820, Israel; 22 National Association of Hungarian Holstein Friesian Breeders, H-1134 Budapest, Hungary; 23 Centre for Genetics, Polish Federation of Cattle Breeders and Dairy Farmers, 00-515 Warsaw, Poland; 24 Department of Genetics and Animal Breeding, Poznan University of Life Sciences, Wolynska 33, 60-637 Poznań, Poland; 25 National Livestock Breeding Center, Nishigo-mura, Fukushima, 961-8511, Japan; 26 Instituto Nacional de Investigación Agropecuaria, 90100 Montevideo, Uruguay; 27 Facultad de Agronomía, Universidad de la República, 12900 Montevideo, Uruguay

## Abstract

A global comparison of dairy cow breeding objectives provides valuable insight into areas of convergence and divergence, helping identify populations with compatible breeding goals for sourcing germplasm to strengthen domestic genetic improvement programs. The objective of this study was primarily to compare the rankings of Holstein-Friesian artificial insemination sires across countries on the total merit index value of each country. The study also examined how different countries present estimates of genetic merit and how frequently they update their base populations; Denmark, Finland, and Sweden (DFS) were treated as a single group. The partial correlation among 22 indexes (i.e., 21 national indexes plus the Holstein Association USA Total Performance Index) was estimated for 49,450 Holstein(-Friesian) sires born post-2000 with a reliability >70%, after adjustment for genetic trends. The partial correlations among the ranking of sires on different indexes varied from 0.24 to 0.87 with 41% of the pairwise correlations being stronger than 0.70 but just 11% being stronger than 0.80. Notably, indexes designed for indoor confinement-based systems had, on average, weaker correlations with grazing-focused indexes from Ireland and New Zealand (0.48 to 0.50). Stronger average correlations (0.69) were observed among the ranking of sires on confinement-based indexes; the correlation between the Irish Economic Breeding Index and New Zealand Breeding Worth was 0.56. When limited to just the milk production components of the different indexes, the partial correlations among the ranking of sires on subindexes were, on average, 0.78, varying from 0.29 (Uruguay with Poland) to 0.96 (Japan with Spain). Countries differ in their approach to trait weighting. Across the 21 countries examined in this study, 38% derived the weights assigned to traits solely from economic models or functions, whereas an additional 29% of the countries adopted only a desired-gains approach. The remainder of countries adopted a hybrid strategy, applying economic values to certain traits, typically the production traits, while using desired gains for others (e.g., the Netherlands, DFS). In some cases, initial weights were derived from economic principles but subsequently adjusted to achieve specific desired gains (e.g., Canada, Australia, Uruguay). Of the 21 milk production subindexes compared, 8 had a negative weight on milk yield (i.e., Canada, DFS, United Kingdom, New Zealand, Australia, Belgium, Ireland, Uruguay), 4 had a positive value (i.e., United States, Poland, Spain, South Africa), whereas Germany, the Netherlands, Japan, France, Slovenia, Italy, Israel, Switzerland, and Hungary did not consider milk yield in their total merit indexes. With the exception of Ireland, United Kingdom, United States, Israel, and Uruguay who presented genetic evaluations of individual animals as PTA, genetic evaluations of milk production traits are presented as EBVs by all other countries. Of the 21 countries, 10% update their genetic evaluation base population more than once per year, 38% update it annually, 38% update it every 5 yr, and the remaining 14% update it periodically. Health traits are increasingly being considered for inclusion in future breeding objectives as are traits associated with both environmental impact and feed efficiency. The findings underscore how economic, biological, genetic, and policy factors shape national breeding objectives, helping interpret international differences in genetic trends and performance outcomes.

## Highlights

• Breeding goals differ widely across countries despite some shared priorities.
• Indexes for grazing systems especially differ from indoor production systems.
• Countries rank top dairy bulls quite differently, reflecting varied systems.
• Approaches to weighting traits vary: economic, desired gains, or a hybrid of both.
• Genetic bases are updated at very different intervals (yearly to >5 years).

## Graphical Abstract

**Figure:**
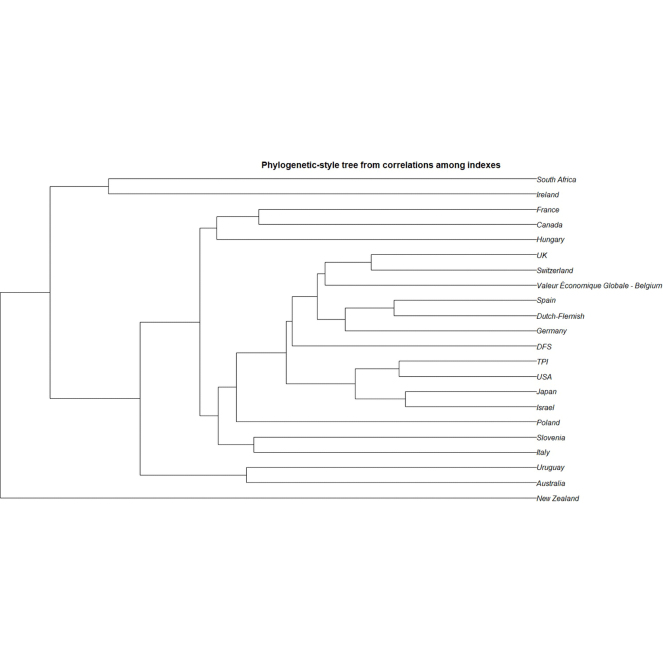
**Summary:**Comparing dairy cow breeding objectives across countries offers a high-level perspective on where national breeding priorities align or diverge. The rankings of 49,450 Holstein-Friesian artificial insemination sires across 22 different dairy breeding indexes differing in component traits, trait weightings, and genetic evaluations were compared; the pairwise correlations between the indexes varied from 0.24 to 0.87, with 41% of the correlations being stronger than 0.70 but just 11% being stronger than 0.80. Of the countries explored, 38% based their trait weightings on economic principles, whereas 29% adopted only a desired-gains approach; the remainder chose a hybrid between economic weights and desired-gains weights. Of the countries in this study, 10% update their genetic evaluation base population more than once per year, 38% update it annually, 38% update it every 5 years, and the remaining 14% update it periodically. DFS = Denmark, Finland, and Sweden; TPI = Holstein Association USA Total Performance Index; UK = United Kingdom; USA = United States of America.

Many population-specific factors influence not just the list of traits considered in a breeding objective but also the relative weights on those traits. A whole myriad of factors influence this decision including, among others, the production systems, climate, regulatory frameworks, the product dairy portfolio and markets (e.g., liquid milk, cheese), the costs of production, trait-specific data availability, covariance components among the traits, and the population mean of each trait. A global comparison of dairy cow breeding objectives provides valuable insights into areas of convergence and divergence, helping identify populations with compatible breeding goals for sourcing germplasm to strengthen domestic genetic improvement programs. Exploring the construction of breeding objectives can also help identify traits and approaches possibly worth considering elsewhere, especially when referencing countries that have experienced strong genetic gain for some traits. Understanding likely future evolution of breeding objectives including new traits of focus helps identify emerging trends across comparable industries. Selection across countries with well-aligned breeding goals should be more favorable than selection just within a country; this is especially true for smaller populations and those with a lower mean performance (Banos and Smith, 1991).

The primary objective of the present study was to compare dairy cow breeding objectives used in different countries. This was achieved by comparing the ranking of high reliability Holstein-Friesian artificial insemination (**AI**) bulls ranked on each index. A further point of interest was the methodology used to determine trait weightings, specifically, whether countries adopted an economics-based approach or a desired-gains framework. Finally, the study explored which traits are being considered for inclusion in future updates of each country's total merit index. Although some countries operate more than one index, this study focused on the most popular index per country.

A total of 21 countries (Denmark, Finland, and Sweden [**DFS**] were treated as one population) participating in Interbull Holstein evaluations were asked to participate in this study; DFS operate an across-country genetic evaluation system with a common breeding objective. Because of its global recognition, the Holstein Association USA (Brattleboro, VT) Total Performance Index (**TPI**) was also included, though only in the correlation analyses, bringing the total number of indexes evaluated to 22. The indexes (country) considered were as follows: Lifetime Performance Index (Canada); Relativ-Zuchtwert Euro (Germany); Nordic Total Merit (DFS); Index de Synthèse Unique (France); Production, Functionality, Type (Italy); Dutch-Flemish Index (the Netherlands and Flanders); Net Merit Index (United States); Index de Sélection Totale (Switzerland); Profit Lifetime Index (United Kingdom); Breeding Worth (New Zealand); Balanced Performance Index (BPI, Australia); Valeur économique globale (Belgium); Economic Breeding Index (Ireland); Indice Combinado (Spain); Plemenska Vrednost 12 (Slovenia); Production Dairy 20 (Israel); Holstein Global Index (Hungary); Economic Index (Poland); Logix Merit Index (South Africa); and Nippon Total Profit (Japan). Respondents were asked which approach they use to decide the emphasis placed on each trait in the index (i.e., economic value, desired gains, or hybrid). If they selected economic value, they were then asked to provide the economic values applied to milk traits. Only milk traits were explored because their definition is constant across jurisdictions. Other questions related to whether EBVs or PTAs were published and whether these were reported on the scale of measurement or as standardized values; a distinction was made between the milk production traits and other suites of traits. How often the genetic base was updated was also asked. Finally, the list of Holstein(-Friesian) AI bulls available on the Council on Dairy Cattle Breeding website (https://webconnect.uscdcb.com/#/top-animal-listing) was circulated to all countries and the total merit index values of these bulls per country with the associated reliability value were requested; each country was asked to add Holstein(-Friesian) bulls missing from this list but >70% reliability in the respective country. A list of 113,498 Holstein(-Friesian) bulls was compiled. Only bulls born from the year 2000 on were considered further. A total of 49,450 were considered in the analysis, albeit not all had an index value in every country. December 15, 2025, was the time point used for the genetic evaluation and index weights. The EBVs or PTAs used were from the official genetic evaluation in the respective country at the time; they were, therefore, likely a combination of genomic, domestic, and multiple across countries evaluations.

Pairs of indexes were analyzed jointly using multivariate ANOVA to account for their correlation and to adjust for systematic effects of year of birth using the model **y***_ij_* = **µ** + yob*_i_* + **e***_ij_*, where **y***_ij_* is the 2 × 1 vector of pair of indexes for the *j*th animal in the *i*th year-of-birth class, **µ** is the vector of overall means, yob*_i_* is the fixed effect class of year of birth, and **e***_ij_* is the residual vector, assumed to follow a bivariate normal distribution with mean zero and an unstructured 2 × 2 residual covariance matrix. The residual correlation matrix among index pairs was obtained from the analysis. In a separate set of analyses, the milk production subindex of the total merit indexes was isolated for those with trait weighting based on economic values; the milk production subindex was the sum of the estimate of genetic merit for each milk trait in that country times its respective economic weight. The partial correlations among these milk production subindexes were calculated using the same approach as used for the total merit index analysis.

Two main approaches are used to decide which weights to associate with individual traits in a breeding objective or selection index—applying economic values or using a desired gains approach. Indexes based on economic values are profit-based, with the weights assigned to traits based on their expected change in profit per unit change in that trait holding all else equal; therefore, the economic value on a trait in an index is a function of, among others, the other component traits in that index. The economic values can be based on current costs and prices, historical costs and prices, or future expected costs and prices. Given the long-term nature of breeding, it should ideally consider future market conditions, production costs, output prices, and any expected potential changes in policy. A desired-gains index, however, provides more control over the trajectory of genetic change, especially for traits with low monetary value but of potentially high (future) strategic importance (e.g., health or welfare). A predefined rate of genetic gain for a set of targets is decided upon, such as improving fertility by a certain percentage and, instead of economic modeling determining the appropriate trait weights, the weights are back-calculated to achieve, where possible, those desired gains. Both approaches are valid, each with their advantages and shortcomings. Economic values can be more defendable assuming that reputable sources of information (e.g., costs, prices) and peer-reviewed approaches are used; they tend to be more objective in nature as opposed to desired gains, which are often based on subjective preferences. Being profit-based also potentially resonates more with the end users, which could lead to greater acceptance and uptake. That said, desired gains can be more easily explained by simply stating, for example, that you want the rate of gain in one trait to be twice that of another trait. Back-calculating the weights for desired-gains indexes is, however, dependent on accurate estimates of the underlying genetic parameters.

Across the 21 countries examined in this study, 38% assigned weights to traits solely from economic models or functions (i.e., Belgium, Germany, Ireland, Israel, New Zealand, Poland, United Kingdom, and United States) and an additional 29% of the countries adopted only a desired-gains approach (i.e., Hungary, Italy, Japan, Slovenia, South Africa, and Switzerland). A subset of countries adopted a hybrid strategy, applying economic values to certain traits, typically the production traits, while using desired gains for others (e.g., Netherlands, DFS, and Spain). In some hybrid cases, initial weights were derived from economic principles but subsequently potentially adjusted to achieve specific desired gains (e.g., Canada, Australia, Uruguay, and France). It is important to note that differences in the weights assigned to individual traits across countries do not necessarily equate to differences in (expected) genetic gain. Along with selection intensity, genetic gain depends not only on the genetic variance of each trait within the population under selection, but also on the covariances among all traits included in the index and their respective relative weighting.

The partial correlations among the ranking of sires on the 22 different indexes are in Table 1. An average-linkage hierarchical clustering on one minus the pairwise correlation distance was applied to the correlation matrix, and the indexes reordered by the dendrogram leaf order. The weighted mean reliability of the sires for the indexes was 0.83.

**Table 1:** Correlations (below the diagonal) and number of sires contributing to those pairwise correlations (above the diagonal) between various national dairy cow total merit indexes^1^

Country	NZ	AUS	URG	ITA	SLO	POL	ISR	JPN	USA	TPI	DFS	GER	NLD	SPA	BEL	SWZ	UK	HUN	CAN	FRA	IRE	SA
NZ		1,224	998	641	556	1,162	1,201	1,241	1,138	1,138	586	877	780	1,211	1,063	1,150	1,427	1,069	571	740	1,058	451
AUS	0.40		1,500	1,813	1,133	3,575	3,532	3,701	3,475	3,475	1,539	2,965	1,022	3,617	3,090	1,421	4,057	3,283	1,697	2,186	2,432	1,043
URG	0.52	0.72		1,590	1,310	4,409	6,007	7,007	7,564	7,564	1,336	2,046	2,041	6,372	3,294	7,239	7,612	6,414	2,427	1,942	1,337	784
ITA	0.39	0.67	0.65		1,410	4,703	4,904	4,989	4,705	4,705	1,860	3,918	2,289	4,863	3,700	5,028	4,587	4,330	1,992	2,890	1,600	932
SLO	0.54	0.51	0.53	0.73		2,304	2,301	2,303	1,990	1,990	1,360	1,685	2,077	2,251	2,286	2,166	2,167	2,142	1,189	1,822	999	658
POL	0.35	0.68	0.54	0.65	0.72		14,320	17,953	15,060	15,060	3,576	10,963	4,963	17,570	11,453	4,541	14,889	16,953	5,940	8,613	3,203	1,470
ISR	0.49	0.54	0.52	0.69	0.69	0.77		21,156	22,334	22,334	3,520	10,289	2,973	20,161	9,811	5,240	19,993	19,666	4,246	6,597	3,177	1,497
JPN	0.55	0.62	0.58	0.74	0.83	0.78	0.87		24,840	24,840	3,510	10,541	5,739	27,300	1,153	5,124	23,882	27,138	7,346	8,616	3,405	1,485
USA	0.61	0.51	0.45	0.51	0.65	0.66	0.80	0.83		36,716	2,800	13,368	1,770	22,337	8,622	5,753	25,094	22,449	7,009	6,057	2,908	1,442
TPI	0.53	0.58	0.51	0.61	0.65	0.67	0.84	0.83	0.87		2,800	13,368	1,770	22,337	8,622	5,753	25,094	22,449	7,009	6,057	2,908	1,442
DFS	0.67	0.67	0.66	0.72	0.66	0.64	0.73	0.80	0.73	0.73		2,962	1,581	3,435	3,150	1,377	3,072	3,184	1,419	2,678	1,390	775
GER	0.60	0.58	0.65	0.67	0.75	0.69	0.80	0.82	0.78	0.77	0.80		3,306	10,160	6,620	4,936	8,555	9,739	2,830	7,763	2,680	1,173
NLD	0.37	0.57	0.58	0.68	0.71	0.73	0.73	0.79	0.71	0.74	0.71	0.81		5,669	3,967	870	2,236	5,565	817	6,257	1,233	461
SPA	0.46	0.68	0.60	0.74	0.80	0.86	0.84	0.87	0.74	0.82	0.74	0.82	0.86		11,246	4,927	22,421	25,371	7,275	8,380	3,322	1,470
BEL	0.60	0.77	0.71	0.75	0.73	0.68	0.72	0.81	0.72	0.77	0.78	0.76	0.78	0.82		2,972	9,529	10,801	4,374	6,338	2,811	1,370
SWZ	0.47	0.70	0.74	0.70	0.62	0.66	0.73	0.76	0.59	0.72	0.73	0.77	0.76	0.80	0.77		4,692	4,540	1,804	5,769	1,222	562
UK	0.44	0.71	0.74	0.70	0.66	0.68	0.72	0.80	0.75	0.75	0.82	0.83	0.81	0.78	0.82	0.84		21,896	7,183	5,904	3,498	1,482
HUN	0.35	0.50	0.43	0.61	0.66	0.70	0.73	0.70	0.60	0.73	0.54	0.62	0.67	0.81	0.72	0.65	0.57		6,653	8,125	3,021	1,372
CAN	0.43	0.64	0.56	0.68	0.67	0.62	0.66	0.69	0.61	0.73	0.67	0.71	0.69	0.78	0.79	0.77	0.73	0.73		1,929	1,205	855
FRA	0.40	0.63	0.61	0.70	0.58	0.60	0.62	0.57	0.51	0.66	0.66	0.69	0.71	0.77	0.66	0.79	0.69	0.65	0.73		2,172	934
IRE	0.56	0.56	0.67	0.51	0.45	0.35	0.36	0.45	0.47	0.44	0.64	0.52	0.51	0.41	0.57	0.61	0.61	0.24	0.50	0.51		750
SA	0.41	0.67	0.53	0.53	0.49	0.58	0.54	0.57	0.48	0.54	0.60	0.57	0.59	0.65	0.56	0.67	0.64	0.41	0.56	0.58	0.59	

1 NZ = New Zealand; AUS = Australia; URG = Uruguay; ITA = Italy; SLO = Slovenia; POL = Poland; ISR = Israel; JPN = Japan; USA = United States of America; TPI = Holstein Association USA Total Performance Index; DFS = Denmark, Finland, and Sweden; GER = Germany; NLD = the Netherlands; SPA = Spain; BEL = Belgium; SWZ = Switzerland; UK = United Kingdom; HUN = Hungary; CAN = Canada; FRA = France; IRE = Ireland; SA = South Africa.

The correlations were adjusted for year of birth of the sire to account for the fact that bulls born later are, on average, higher on both indexes, which can inflate the correlations because they can reflect shared time effects rather than true ranking consistency. What remains following adjustment for the genetic trend is the within-cohort ranking, which provides a truer measure of cross-country consistency. The partial correlations among the ranking of sires on the indexes were, on average, 0.20 weaker across all pairwise correlations compared with the raw correlations. However, when restricted to a 5-yr period (i.e., bulls born between 2005 and 2009) the raw correlation differed from the partial correlation by, on average, just 0.01 with some correlations being weaker than the year adjusted partial correlations. Hence, it is important to adjust for genetic trends when comparing breeding indexes, especially where the rate of genetic gain might differ per population.

The partial correlations among the ranking of sires on all 22 indexes varied from 0.24 (between Ireland and Hungary) to 0.87 (between Israel and Japan); the average correlation was 0.65. The lack of a perfect correlation between pairs of indexes can be due to differences in both the genetic evaluations themselves, including the mean sire reliability, but also the relative weights on the individual traits and what traits actually are included in the index. In the case of economics-based indexes, the weights on the individual traits are a function of both the value of the outputs but also the costs of production; in the case of desired gain or hybrid indexes, the weighting factors per trait are a function of the desired expected rate of change in each trait as well as the covariance components of all traits within the index. Differences in the traits included in the indexes of various dairy cow breeding objectives and their relative emphasis have been reported elsewhere (Miglior et al., 2005; Cole and VanRaden, 2018).

New Zealand's Breeding Worth and Ireland's Economic Breeding Index were, on average, the least correlated with the other indexes, each with a mean correlation between sire rankings of 0.50 and 0.48, respectively, across all pairwise comparisons. The correlation between sire rankings on the Breeding Worth and Economic Breeding Index was 0.56. The TPI was also included because of its global recognition. Across all countries, the average correlation with TPI was 0.68, ranging from 0.44 with Ireland's Economic Breeding Index to 0.87 with the US Net Merit Index. The ranking of sires on the TPI was, on average, more correlated with other indexes than the Net Merit Index (USA) with the strength of the correlation being stronger for the former almost 3 times more often. Among the indexes compared, the Logix Merit Index of South Africa was the most consistent in its correlation with other indexes, whereas the Holstein Global Index of Hungary was the most variable in its correlations.

To examine the relationships among indexes in more detail, a milk production subindex was created for each population using only milk production traits; correlations among the ranking of sires on these subindexes are in Table 2. The average partial correlation between milk production subindexes was 0.78, ranging from 0.29 (Uruguay and Poland) to 0.96 (Japan vs. Spain). These milk-subindex correlations were stronger than the correlations between the corresponding total merit indexes in 85% of pairwise comparisons, exceeding them by an average of 0.10. Even so, the correlations were not perfect, reflecting differences in (co)variance components and trait weights, including zero weights for traits not represented in a given subindex. Among the 21 milk production subindexes, 8 assigned a negative weight to milk yield (Canada, DFS, New Zealand, United Kingdom, Australia, Belgium, Ireland, and Uruguay), 4 assigned a positive weight (United States, Poland, South Africa, and Spain), and Germany, Japan, Slovenia, Italy, the Netherlands, France, Israel, Switzerland, and Hungary did not explicitly weight milk yield. The economic value of protein relative to fat, derived from national economic models, varied from 0.56 (Canada) to 3.16 (Ireland), with a mean of 1.60. Fat and protein concentrations were explicitly included in the total merit indexes of Italy, Slovenia, and France, and protein concentration was included in the Index de Sélection Totale (ISET) index of Switzerland; lactose concentration was included in the Relativ-Zuchtwert Euro (RZEuro) index of Germany. Despite these differences, variation in sire rankings on the milk subindex (for the 8 countries using economic values) was driven mainly by the underlying genetic evaluations rather than the economic weights. Using country-specific milk-trait economic values applied to Irish PTA increased the mean partial correlation among milk production subindexes from 0.76 to 0.92. Deterministic estimates based on Irish genetic covariance components suggested the expected correlations should average 0.97. This indicates that differences in (co)variance components may contribute to imperfect agreement between indexes more than the trait weighting per se; for example, the correlation between the US Net Merit milk subindex calculated using US versus Irish genetic evaluations was 0.84. Therefore, consistent with earlier research, modest changes in trait weights generally have limited impact on overall total merit rankings (Weller, 1994).

**Table 2:** Correlations among sire rankings for the milk production components of the various national dairy cow total merit indexes^1^

Index	URG	NZ	SA	USA	HUN	POL	FRA	ISR	ITA	SPA	JPN	GER	NLD	SWZ	UK	AUS	IRE	DFS	CAN
NZ	0.58																		
SA	0.31	0.53																	
USA	0.45	0.67	0.86																
HUN	0.45	0.63	0.81	0.90															
POL	0.29	0.48	0.79	0.79	0.75														
FRA	0.63	0.63	0.71	0.78	0.71	0.76													
ISR	0.40	0.49	0.89	0.90	0.88	0.86	0.81												
ITA	0.56	0.57	0.73	0.77	0.71	0.83	0.86	0.90											
SPA	0.32	0.54	0.88	0.90	0.86	0.93	0.86	0.93	0.95										
JPN	0.46	0.61	0.85	0.88	0.83	0.88	0.85	0.92	0.93	0.96									
GER	0.52	0.65	0.81	0.84	0.78	0.83	0.85	0.89	0.87	0.92	0.91								
NLD	0.51	0.62	0.75	0.85	0.80	0.82	0.82	0.89	0.85	0.92	0.93	0.93							
SWZ	0.61	0.64	0.81	0.84	0.78	0.82	0.90	0.91	0.90	0.89	0.93	0.92	0.90						
UK	0.62	0.61	0.76	0.85	0.80	0.76	0.86	0.87	0.86	0.84	0.89	0.88	0.89	0.93					
AUS	0.68	0.80	0.64	0.75	0.71	0.58	0.74	0.60	0.70	0.58	0.64	0.70	0.69	0.76	0.73				
IRE	0.79	0.65	0.56	0.67	0.61	0.58	0.81	0.50	0.77	0.51	0.61	0.75	0.72	0.80	0.63	0.79			
DFS	0.64	0.67	0.68	0.80	0.74	0.73	0.85	0.82	0.83	0.83	0.87	0.86	0.83	0.86	0.86	0.77	0.82		
CAN	0.36	0.42	0.57	0.92	0.58	0.83	0.66	0.63	0.68	0.62	0.66	0.91	0.67	0.61	0.62	0.82	0.81	0.88	
BEL	0.69	0.73	0.74	0.86	0.83	0.71	0.79	0.80	0.79	0.54	0.88	0.84	0.83	0.87	0.89	0.85	0.82	0.85	0.93

1 URG = Uruguay; NZ = New Zealand; SA = South Africa; USA = United States of America; HUN = Hungary; POL = Poland; FRA = France; ISR = Israel; ITA = Italy; SPA = Spain; JPN = Japan; GER = Germany; NLD = the Netherlands; SWZ = Switzerland; UK = United Kingdom; AUS = Australia; IRE = Ireland; DFS = Denmark, Finland, and Sweden; CAN = Canada; BEL = Belgium.

Of the 21 countries, 10% update their genetic evaluation base population more than once per year, 38% update it annually, 38% update it every 5 yr, and the remaining 14% (i.e., Ireland, New Zealand, and Slovenia) update it less often than every 5 yr. Updating the genetic evaluation base population has both advantages and disadvantages. One advantages of a (frequent) base update is that it addresses the fact that breeders and producers often view very high index values as unrealistic. When the base is recent, genetically superior animals receive favorable (often positive) estimates of genetic merit, whereas genetically inferior animals receive unfavorable estimates. Although breeders can find it challenging to market animals with negative genetic evaluations or index values, this distinction can actually encourage the use of the most genetically elite animals.

Disadvantages of frequent base updates include the difficulty breeders and producers may experience in assessing long-term genetic progress within their own herds; regular updates may, however, make benchmarking against the national population easier. When a base is not updated regularly, a subsequent update can cause large shifts in estimates of genetic merit (and indexes), particularly following periods of rapid genetic improvement. Such shifts may create confusion or frustration among breeders and producers, underscoring the need for a strong communication plan. Many breeders and producers use independent culling thresholds for some traits such as direct calving ease or calving difficulty. They rely on rule-of-thumb fixed threshold values to avoid calving complications. A change in base requires these thresholds to be updated in the minds and practices of producers and breeders, and a failure to recalibrate, especially following a period of strong genetic gain, may have serious consequences.

With the exception of DFS, all other countries report estimates of genetic merit for the milk production traits on their unit of measurement which, for the yield traits, is kilograms; the exception is the United States where yield is measured and expressed in pounds. Ireland, United Kingdom, United States, Israel, and Uruguay publish PTA, which represent half of the EBV values that are published by all other countries. Predicted transmitting abilities for dairy bulls are often preferred by producers because they directly reflect what daughters are expected to inherit from the sire, making both individual trait values and profit-based indexes immediately interpretable. For geneticists, however, EBVs are more intuitive, as they relate directly to the additive genetic variance and other genetic parameters. The EBVs are also more appropriate when examining genetic trends in females because it is the EBV, rather than the PTA, that the cow herself expresses. Although the majority publish estimates of genetic merit for the production traits on their unit of measure, the same was not true for the genetic evaluations of all other traits with many countries using standardized values. The mean and SD of the transformed estimates of genetic merit varied considerably across countries. For individual traits, some countries use a SD of 1 (e.g., France), 5 (e.g., Canada), 10 (e.g., Belgium), or 12 (e.g., Germany, South Africa). Having a different SD can make comparison across countries challenging. For example, assuming a SD of 5, then an animal with a score exceeding 10 is expected to be in the top 2.2% of the population; this animal is only in the top 20% of the population if the actual SD is 12.

Disease and health traits, especially those related to feet and legs, were most frequently cited as priorities for inclusion in breeding objectives in the near future. Ten countries referred to health-related traits, including leukosis (Canada) and Johne's disease (Ireland and United Kingdom). Methane emissions were flagged by 9 countries, and 6 highlighted traits related to feed intake or efficiency, such as DMI, rumination time, feed efficiency, and cow live weight. Several countries also singled out the interval from calving to first insemination as a trait of interest. Overall, these responses reflect a general shift in emphasis from production traits to nonproduction traits. It should be noted, however, that some countries may already have evaluations for these traits as well as possibly also have them included in their breeding objective. As genetic trends for reproductive performance improve (García-Ruiz et al., 2016), cow longevity may improve, placing a greater strain on the health and immune system of the cow necessitating corrective action from breeding. At the same time, growing pressure to reduce the environmental footprint of dairy production systems is strengthening the case for genetic selection targeting both enteric methane emissions and feed efficiency.

In conclusion, substantial heterogeneity exists across countries in how dairy breeding objectives are defined, weighted, and expressed, with the divergence among the breeding objectives reflecting differences in economic assumptions, desired genetic trajectories per trait, genetic parameters, and genetic evaluation systems. Convergence was most evident among indexes sharing similar production environments and economic assumptions, whereas divergence arises where breeding objectives are shaped by contrasting production systems, policy constraints, and strategic priorities for long-term sustainability. In practice, however, the degree of alignment between recommended breeding index and the criteria actually used by dairy producers or semen companies may vary, particularly where semen marketing is influenced by the genetic merit of bulls in their country of origin rather than their ranking within national evaluation systems.
